# A “Papageno” Story Interview Suicide Prevention Intervention for Young Adults With Past-Month Suicidal Ideation: Uncontrolled Single-Group Pilot Study of Feedback and Acceptability

**DOI:** 10.2196/71368

**Published:** 2025-10-23

**Authors:** Jane Harness, Kamara Gardner, Jessica L Schleider, Cheryl A King

**Affiliations:** 1 Department of Psychiatry University of Michigan—Ann Arbor Ann Arbor, MI United States; 2 Department of University Health and Counseling University of Michigan Ann Arbor, MI United States; 3 Department of Medical Social Sciences Northwestern University Feinberg School of Medicine Chicago, IL United States

**Keywords:** youths, suicide prevention, Papageno interview, intervention, digital media, narration

## Abstract

**Background:**

Suicide continues to be a leading cause of death for young people, and the 2024 US National Strategy for Suicide Prevention has called for innovative approaches to suicide prevention. This strategy acknowledges the potential of the “Papageno” effect or the suicide preventive effect of stories of people who overcome suicide crises

**Objective:**

This pilot study’s objectives were to develop filmed interviews of young adults sharing their personal story of overcoming suicide crises (“Papageno” stories) and to examine their acceptability and appropriateness in a sample of young adults with a past-month history of suicidal ideation.

**Methods:**

In total, 6 filmed interviews (5 “Papageno” story interviews and 1 active control interview) were videotaped. Study participants were recruited from Instagram and community organizations. Interviewees and participants were between the ages of 18 and 24 years. To be eligible for the study, participants responded “yes” to the question, “In the past 1 month, have you wished you were dead or wished you could go to sleep and not wake up?” Participants provided demographic information and responded to a Brief Mood Introspection Scale. They were subsequently instructed to watch the video, which was followed by the Brief Mood Introspection Scale and questions about the acceptability and helpfulness of the video. They were also asked 5 free-response questions regarding their likes, dislikes, suggestions, and overall reactions to the filmed interviews and whether they would watch it again.

**Results:**

We collected 3-4 participant responses per video for each of the 5 different interview videos (a total of 16 responses). The videos were found to be generally acceptable and appropriate, and no participants reported feeling less likely to reach out for help when needed. Qualitative feedback yielded recommendations to shorten the length of the videos as well as to include multiple interviewees in future videos so that the audience has a better chance to identify with the interviewee.

**Conclusions:**

Filmed “Papageno” interviews were rated as generally acceptable and appropriate, reinforcing the need for more research examining their potential effects on proximal risk factors for suicide. Next steps include the incorporation of feedback to create a singular finalized video and a randomized controlled trial assessing the effects of the Papageno video.

## Introduction

In 2022, suicide was the second leading cause of death for 20- to 24-year-old people and the third leading cause of death for 15- to 19-year-old people in the United States [1]. Public health suicide prevention approaches have involved the creation of guidelines for news and entertainment media due to known suicide contagion or social transmission effects. These guidelines [2-4] advise against showing suicide methods and recommend the encouragement of help-seeking and promotion of crisis resources. Suicide protective effects can occur when alternatives to suicide are modeled and shared in news media [5,6]. This protective effect is often referred to as the “Papageno effect” [7]. It is well known that protective factors are an important part of a suicide risk assessment and that an individual’s reasons for living may modulate the threshold for acting on suicidal thoughts [8,9].

The White House introduced a National Strategy for Suicide Prevention in 2024 [10] due to the ongoing public health concern of suicide. Goal 1 of this plan (Establish effective, broad-based, collaborative, and sustainable suicide prevention partnerships) describes success in this domain as “foster[ing] creativity, new ideas and perspectives.” Furthermore, they suggested collaborating with new industries including entertainment media. Goal 7 (Implement research-informed suicide prevention communication activities in diverse populations using best practices from communication science) referenced the “Papageno” effect in highlighting the potential beneficial effect of safe messaging strategies. For example, they mentioned Logic’s song “1-800-273-8255,” which prompted an increase in calls to the National Suicide Prevention Lifeline and a decrease in suicides around that time [11]. Objective 7.4 is “communicate stories of help, hope and healing using safe messaging strategies,” and objective 7.6 is “engage news media, the entertainment industry, and schools of journalism and mass communication to encourage safe, accurate and responsible ... depictions of suicide and positive mental health coping skills.” In summary, national leadership has acknowledged the urgent need for creative collaborations including leveraging the Papageno effect in suicide prevention strategies.

The literature about the effect of Papageno stories is mostly limited to news reporting [12] but suggests that sharing stories about crisis intervention may promote help-seeking behaviors in viewers [6]. However, one study of fictional “Papageno” stories in entertainment media included an experimental study of viewer response to *Elizabethtown* (2005), which includes a Papageno story. Compared with the 2 other movies that viewers were randomized to in this study (*Night Mother*—protagonist dies by suicide and *A Single Man*—protagonist dies from a heart attack), *Elizabethtown* was found to have a positive impact on viewer’s life satisfaction in the group with lower baseline reasons for living [13]. However, this movie does not have a strong, intentional message of suicide prevention; thus, it remains possible that preventive effects could be amplified with a more targeted message. In keeping with social learning theory [14], which asserts that behaviors are emulated by observers, observing people overcome suicide crises may bring about those same coping, help-seeking, and problem-solving behaviors in viewers.

Furthermore, peer testimonial programs show promise as a suicide prevention strategy. For example, the “Sources of Strength” taped testimonials and accompanying curriculum improved youth knowledge and attitudes about suicide [15]. Participation in this multicomponent program was also associated with lower rates of suicide attempts at 3 months after the program, and youth participants adopted more favorable attitudes toward getting help for themselves or their friends [16]. Seeking help from adults at school was viewed as more acceptable and potentially helpful after participation in the “Sources of Strength” peer-led suicide prevention program compared to a waitlist control [17]. When peer-written testimonials of “hope, help, and strength” were delivered to a target population via text through the Stories of Personal Resilience in Managing Emotions program, they were found to be relatable, useful, intriguing, and likeable [18].

Scalable and accessible online programs, such as single session interventions (SSIs), have shown promising effects for youth mental health in spaces where youths may otherwise have not received any care at all [19]. An SSI including testimonials of older youths who shared their experience of using a growth mindset to cope during adversity found significant improvements in depression and anxiety symptoms compared to the control program as far out as 9 months after the session [19]. During the COVID-19 pandemic, when adolescents were randomized to a behavioral activation SSI, a growth mindset SSI, and supportive control, those randomized to the SSI conditions had decreased hopelessness after the intervention and 3 months after the intervention compared to those randomized to the supportive control [20]. Furthermore, social media users aged 13-44 (average age 17.93, SD 4.92) years flagged as being potentially in crisis who engaged in a 1-minute crisis response SSI had a greater decrease in hopelessness and increased likelihood of using the crisis resources provided compared to users who received the typical crisis response [21]. Online SSIs like this may fill an important gap for people who are on waiting lists for professional mental health care, and they have been shown to be helpful in decreasing hopelessness and increasing help-seeking.

Although suicide prevention organizations have produced hope-inspiring interviews with people who have come close to suicide [22,23], some of these publicly available videos have not been studied for their effects on viewers. For the public-facing videos that have been studied, the results are video-dependent. In a study comparing a music video called “Boys Don’t Cry” to an excerpt from a documentary showing an individual being taught to increase his cognitive processing speed while learning table tennis, both videos had a positive effect on help-seeking intentions among Australian men (age ranging from 18 to 87 years). In a double-blind randomized controlled trial (RCT) in Austria of the “It Gets Better” videos of lesbian, gay, bisexual, transgender, and queer+ individuals overcoming challenges, researchers found small improvements in suicidal ideation for transgender and nonbinary individuals (age ranging from 14 to 22 years) [24]. They also found improvement in help-seeking intentions in the intervention group (compared to the active control video about a healthy lifestyle). Another recent study demonstrated positive effects in Austria. When adolescents, ages 14-19 years, were randomized to watch a video of a 17-year-old telling his story of finding help versus an active control, those who watched the intervention video experienced a decrease in suicidal ideation and improvement in help-seeking intentions [25]. The study of similarly scalable and accessible story-based interventions in the United States for the young adult age group is needed. Additionally, further study of videos created specifically to highlight “Papageno” stories is needed. As such, we set out to film interviews with young adults with “Papageno” stories to determine their acceptability among a group of young adults with a past-month history of suicidal ideation. The social learning theory informs the intervention design because viewers may emulate the help-seeking behavior the interviewee used.

## Methods

### Ethical Considerations

An umbrella institutional review board (IRB) was approved for this project (HUM00208715). Creation of the filmed videos was exempted by the IRB (HUM00223570). The piloting of the filmed videos was approved by the IRB (HUM00230451). Informed consent was obtained for all participants as outlined below. Measures were taken to maintain privacy and confidentiality. Any identifying information needed for compensation purposes was collected in a survey separate from the consent and separate from the study questions. Participants were aware of the compensation, and this information was also included in the consent.

### Intervention

We filmed 5 interviews of young adults, ages 18 to 24 years, with personal histories of “Papageno” stories. Interviewees were recruited from community organizations and met with the principal investigator (PI; JH) at least once to discuss the study and their stories before the filmed interview. They were given information related to how to safely share their story in accordance with the guidelines. They were created with the purpose of this research study and are not currently publicly available. Each interviewee was asked questions about what helped them during a time in their life when they came close to suicide. Additionally, the interviews focused on recovery, help-seeking, reasons for living, and hope for the future. Example follow-up questions include: “What was helpful during that time?” “What advice would you give to your younger self?” and “What advice would you give to people watching this video?” Interview questions were tailored in real time to guide the interviewee in telling their “Papageno” story and to highlight what was distinctly helpful to them during that time. Because each of their stories was unique, we did not use a standardized set of questions. A TIDieR (Template for Intervention Description and Replication) table outlining key components of the intervention can be found in Multimedia Appendix 1. An active control educational video about mental health resources was also created. All interview videos were between 20 and 35 minutes, and the role of the interviewer was to elicit the interviewee’s Papageno story of hope and recovery. The visuals sometimes included shots of 2 people talking in a room, and at other times, the camera is focused solely on the interviewee. All of the videos followed the same structure of the individual first sharing their “Papageno” story and then discussion of what was helpful for them. Videos were filmed by a professional and edited by the PI. Video length was determined by the time needed for each interviewee to share their story and what was helpful during that time. Videos followed all guidelines for media with suicide content including not discussing suicide methods. Participants could have completed the survey at any point after watching the video, but they were encouraged to set aside time to do it all together in 1 sitting if possible.

### Recruitment

Participants were recruited from social media (specifically Instagram) through targeted advertisements and mental health community groups via email. Inclusion criteria were ages 18-24 years and a past-month history of suicidal ideation. An exclusion criterion was the inability to participate in an online Qualtrics survey in English. For those recruited through social media, the advertisement was linked to a Qualtrics survey in which participants could read more about the study. If still interested, they could choose a time to review the consent over Zoom (Zoom Video Communications) with the PI. For those recruited from community groups, potential participants were given the same study information and directed to email the PI if they were interested in scheduling the consent process. During the Zoom meeting, the PI asked the participant two yes or no questions to ensure eligibility: (1) Are you between the age of 18 and 24 years? (2) In the past 1 month, have you wished you were dead or wished you could go to sleep and not wake up?

If the participant answered yes to both questions, the PI shared the link to the Qualtrics consent form via chat. The consent form was reviewed in real time together over Zoom, and the participant was given a chance to ask questions. After the participant verbally and electronically consented, they were sent a separate Qualtrics link to the study survey with the embedded video. Participants were compensated US $20 in the form of an e-gift card for each video that they reviewed for up to 5 videos. If participants wanted to respond to more than 1 video, they emailed the PI for the next video’s Qualtrics link. Crisis numbers were provided during the recruitment process and in the study survey. Because participants needed to email the PI for the link to the next video survey, there was necessarily at least some time in between the viewings. Participants did not choose which video they received. There was no way for us to determine if the participants watched the full video with full attention, but they could not advance to the next survey page until at least half of the video length time had elapsed in case they decided to watch it at 2 times the original speed.

### Measures

To test the viewer’s mood before and after watching the filmed interview, a single-item question adapted from the Brief Mood Introspection Scale [26] was used. Participants were asked, “Overall my mood right now is –10=very unpleasant to 10=very pleasant.” The rationale for including this scale was to capture any unintended induced dysphoria that could co-occur with improvements in other measures such as hope for the future and help-seeking intentions.

To test filmed interview acceptability, the Program Feedback Scale (PFS) [27] and Acceptability of Intervention Measure (AIM) [28] were used. The PFS asks participants to rate agreement with 7 statements (eg, “I enjoyed the filmed interview”) on a 5-point Likert scale (1=strongly disagree and 5=strongly agree). They were also asked the open-response questions: “What did you like about the filmed interview?” and “What would you change about the filmed interview?” The PFS was created to test SSIs and adapted from validated assessments intended for digital interventions [29]. The AIM asks participants to rate agreement with 4 statements (eg, “The filmed interview meets my approval”) on a 5-point Likert scale (1=strongly disagree and 5=strongly agree).

To test filmed interview appropriateness, the Intervention Appropriateness Measure (IAM) [28] was used. The IAM asks participants to rate agreement with 4 statements (eg, “The filmed interview seems applicable”) on a 5-point Likert scale (1=strongly disagree and 5=strongly agree). The AIM and the IAM have good structural validity and reliability, and each measure is sensitive to change in both directions.

Participants were asked the following 3 questions, with response options rated on a 5-point Likert scale (1=significantly less hopeful or understood or likely and 5=significantly more hopeful or understood or likely).

“Compared to before you watched this filmed interview, are you feeling” (1) more or less hopeful about the future? (2) more or less understood? or (3) more or less likely to reach out for help in the future?

Participants were prompted with the following to collect open-ended responses: (1) What did you like about the filmed interview? (from the PFS) (2) What would you change about the filmed interview? (from the PFS) (3) Please provide any additional comments or suggestions for us related to the filmed interview you watched. (4) What are your overall reactions to this filmed interview? (5) Would you watch this video again on your own time if you were going through a hard time? Why or why not?

Finally, to test participant identification with the interviewee, 8 items from Cohen Identification Scale [30] were used. Examples of items on this scale include: “I think I have a good understanding of the interviewee” and “While viewing the interview, I could feel the emotions the interviewee portrayed.” Answers are rated on a 5-point Likert scale (1=strongly disagree and 5=strongly agree).

### Statistical Analysis

We collected both quantitative and qualitative data. Because our sample was small (N=16 for the interview videos), we limited our analyses to descriptive statistics. To analyze the open-ended responses, we used content analysis [31] to highlight frequencies of codes for each of the 5 free-response questions. Authors JH and KG used an inductive approach and memoed to create a list of themes that were then consolidated into the final codebook through real-time consensus. Using the codebook, authors JH and KG coded each participant’s response to each question. Where there were disagreements in coding, authors JH and KG met to reconcile the differences in real time. The codes and their frequencies as well as a representative quote were compiled to summarize the main findings of the qualitative data.

## Results

Sample demographic characteristics can be found in Table 1. Because participants were able to provide feedback for up to 5 videos, the demographic information is reported per response for the 16 total responses for the intervention (Papageno story interview) videos. The sample size, therefore, was smaller than the number of responses. One video had 4 responses, and the remaining 4 videos had 3 responses each. All of the results we describe here must be interpreted in the context of having a very small sample size, which prohibits statistical testing; therefore, no meaningful effects can be assumed. Participants reported an average pre- to postmood change of +2.3 (SD 5.9; scale ranging from –10=very unpleasant to 10=very pleasant). For all of the questions involving a 5-point Likert scale (1=strongly disagree and 5=strongly agree), the averages of the responses were calculated and can be found in Table 2. For the measures of acceptability (the PFS and the AIM), response averages ranged from 3.75 to 4.5 (SD ranging from 0.48 to 1.18). For the measure of appropriateness (the IAM), response averages ranged from 3.94 to 4.13 (SD ranging from 0.44 to 0.77). For the measures of identification with the interviewee (questions from Cohen’s Identification Scale), response averages ranged from 3.06 to 4.19 (SD ranging from 0.54 to 1.18). There were 3 instances of missing responses for the quantitative questions: 1 postintervention mood scale, 1 response to the question about whether the filmed interview would help other people their age, and 1 response to the question about whether they felt as if they were there while watching the filmed interview. Because missing data were rare, and this is an uncontrolled pilot study, we performed the statistical analyses without adjusting for the missing data.

Results regarding changes in future hopefulness, feeling understood, and the likelihood to reach out for help in the future are reflected in Table 3. No participants reporting feeling less likely to reach out for help in the future. In total, 4 responses indicated feeling somewhat less understood, but there were 0 responses for feeling significantly less understood. There was 1 response for feeling significantly less hopeful about the future, but this particular respondent also reported feeling significantly more likely to reach out for help in the future and said “I will love to watch more videos” when asked to provide additional comments or suggestions, showing that this response may have been an error. There were 0 responses for feeling somewhat less hopeful about the future.

Qualitative results for the 5 open-ended questions are recorded in Tables 4-8. When asked what they liked about the filmed interview, participants’ responses were coded with the following codes (from most frequent to least): hopeful or helpful, knowledge, genuine, relatable or understandable, comprehensive, and simple. When asked what they would like to change about the filmed interview, 7 of the 16 responses were coded as “nothing.” The most frequent recommendation was to shorten the length of the filmed interviews, with one respondent noting: “... Especially when I am at my lowest emotional points I do not think I would watch a 33 minute interview” and to include more stories or perspectives (n=4, 21.1% for each). In total, 3 responses had specific suggestions for the interviewer. One person asked for captions (which were available to be turned on, but they may have missed the button). When asked for additional comments or suggestions, 6 (46.2%) responses were coded as asking for more stories and perspectives. Additionally, the length of the videos came up again (n=2, 15.4%). A total of 2 responses were coded as “hopeful or helpful,” and 2 responses had specific advice about the visual elements of the filmed interview. Finally, 1 person mentioned again the desire for subtitles.

**Table 1:** Respondent characteristics (N=16).

Characteristics		Values
**Age (years)**		
	Mean (SD)	22.3 (1.7)
	Range	18-24
**Sex, n (%)**		
	Male	3 (18.8)
	Female	10 (62.5)
	Nonbinary	2 (12.5)
	Transgender male	1 (6.3)
**Ethnicity, n (%)**		
	Hispanic	0 (0)
	Non-Hispanic	16 (100)
**Race, n (%)**		
	Asian	5 (31.3)
	Black or African American	6 (37.5)
	White	5 (31.3)

**Table 2:** Average scores for participant identification with the interviewee, filmed interview appropriateness, and filmed interview acceptability.

			Average (SD)^a^
**Program Feedback Scale**			
	I enjoyed the filmed interview	3.94 (0.57)	
	I understood the filmed interview	4.50 (0.52)	
	Watching the filmed interview was easy to do	4.13 (0.72)	
	I was well-focused when I watched the filmed interview	4.06 (0.57)	
	I think the filmed interview would be helpful to other people my age	3.93 (0.88)	
	I would recommend this filmed interview to a friend going through a hard time	3.75 (1.18)	
	I agree with the filmed interview’s message	4.31 (0.48)	
**Acceptability of Intervention Measure**			
	The filmed interview meets my approval	3.93 (0.59)	
	The filmed interview is appealing to me	3.81 (0.83)	
	I liked the filmed interview	4.06 (0.57)	
	I welcome the filmed interview	4.31 (0.60)	
**Intervention Appropriateness Measure**			
	The filmed interview seems fitting	4.06 (0.68)	
	The filmed interview seems suitable	4.06 (0.44)	
	The filmed interview seems applicable	4.13 (0.62)	
	The filmed interview seems like a good match	3.94 (0.77)	
**Cohen Identification Scale**			
	While viewing the filmed interview, I felt as if I was there	3.13 (1.06)	
	While viewing the filmed interview, I forgot myself and was fully absorbed	3.06 (1.18)	
	I was able to understand the events described by the interviewee in a manner similar to that in which the interviewee understood them	4.00 (0.82)	
	I think I have a good understanding of the interviewee	4.13 (0.62)	
	While viewing the interview, I could feel the emotions the interviewee portrayed	4.19 (0.75)	
	During viewing, I felt I could really get inside the filmed interviewee’s head	4.00 (0.73)	
	At key moments in the interview, I felt I knew what the interviewee was going through	4.19 (0.54)	
	While viewing the interview, I wanted the interviewee to pursue their dreams	4.31 (0.60)	

^a^1=strongly disagree to 5=strongly agree.

**Table 3:** Number of responses for “Compared to before you watched this filmed interview, are you feeling ...”

		Respondents, n (%)
**Level of change in hope about the future**		
	Significantly less hopeful about the future	1 (6.3)
	Somewhat less hopeful about the future	0 (0)
	The same amount of hopefulness about the future	7 (43.8)
	Somewhat more hopeful about the future	7 (43.8)
	Significantly more hopeful about the future	1 (6.3)
**Level of change in feeling understood**		
	Significantly less understood	0 (0)
	Somewhat less understood	4 (25)
	No change in whether you feel more or less understood	2 (12.5)
	Somewhat more understood	7 (43.8)
	Significantly more understood	3 (18.8)
**Level of change in likelihood to reach out for help when needed**		
	Significantly less likely to reach out for help when needed	0 (0)
	Somewhat less likely to reach out for help when needed	0 (0)
	No change in the likelihood of reaching out for help when needed	4 (25)
	Somewhat more likely to reach out for help when needed	9 (56.3)
	Significantly more likely to reach out for help when needed	3 (18.8)

**Table 4:** Content analysis of qualitative data: what respondents liked about the filmed interview.

What did you like about the filmed interview?	Values, n (%)	Representative quote
Hopeful or helpful (effective or encouraging or engaging)	6 (24)	“I liked the fact that it the survivor does not just lament and unleash all her difficult moments as that would have affected my mood, but she went ahead to say how she battled [with it] to cope and thereby dropping some strong words of encouragement signifying [that I] shouldn’t give up or let it way me down.”
Knowledge (crisis resources or examples of ways people have found help)	5 (20)	“This film would not only give a person and advice, but hope as the message itself is coming from a Victim/survivor which makes it more realistic and the messages itself hopeful.”
Genuine (candid or open or emotional or sincere)	5 (20)	“I appreciated how candid the interviewee was. His openness was palpable and it was nice to see how much better he was doing since his attempt. His messages at the end particularly stood out as being some of the interview’s most emotional moments.”
Relatable or understandable	5 (20)	“I found the interviewee to be relatable in her feelings and the way she described them!”
Comprehensive (detailed or holistic)	3 (12)	“I like that both systemic and personal barriers were addressed and the message that things are in flux and the environment influences a lot of how we feel.”
Simple	1 (4)	“It was simple, understandable and effective.”

**Table 5:** Content analysis of qualitative data: what respondents would change about the filmed interview.

What would you change about the filmed interview?	Values, n (%)	Representative quote
Nothing	7 (36.8)	“I don’t think there is something to change.”
Too long	4 (21.1)	“I did think the filmed interview was a bit long ...”
More stories or perspectives (not relatable or generalizable, include multiple people, include multiple reasons for living)	4 (21.1)	“Maybe more variety rather than just emphasis on personal relationships. When I am feeling very low, I’m convinced that nobody cares about me, so I don’t think that alone could bring me back.”
Interviewer (less and better questions or prompts)	3 (15.8)	“I feel like the parts of the interview that talked about suicide prevention advocacy took away from the individual story in a way, like it was performative, even though I know it’s not. When I’m feeling bad, I think the personal stories are so much more impactful than hearing about efforts to keep lots of people alive.”
Accessibility needs (captions)	1 (5.3)	“Captions would help because the sound was a little low.”

**Table 6:** Content analysis of qualitative data: additional comments or suggestions from respondents.

Please provide any additional comments or suggestions for us related to the filmed interview you watched.	Values, n (%)	Representative quote
More stories or perspectives (not relatable or generalizable, include multiple people, include multiple reasons for living)	6 (46.2)	“Maybe the speaker could talk about how they brought up the topic of suicide/mental health with their social circles.”
Too long	2 (15.4)	“To condense my previous suggestions, I think a shorter interview with a wider or more relatable breadth of experiences would be a lot more moving and beneficial.”
Hopeful or helpful (effective or encouraging or engaging)	2 (15.4)	“I liked the whole journey story. Hearing about her struggles helped rationalize her solutions.”
Visual (more visuals or videos add graphics or items of visual interest, camera focus)	2 (15.4)	“I would recommend a stronger camera focus on the individual, rather than two people in a room talking; individualizing the interviewee and emphasizing her common ground and character in relation to the viewer is important. I did not get much of a glimpse of her personality, which made the video seem impersonal.”
Accessibility needs (captions)	1 (7.7)	“Providing subtitles would make it more accessible.”

**Table 7:** Content analysis of qualitative data: respondents’ overall reactions.

What are your overall reactions to this filmed interview?	Values, n (%)	Representative quote
Positive outcome or emotion (liked it or good or grateful or less impulsive or more reflective)	8 (34.8)	“My reactions were, for the most part, appreciative of the central message! His advice at the end stuck out to and touched me the most.”
Wish for specific things to be different (content, ambiguity of religious lens, longing for more depth)	5 (21.7)	“I don’t feel happier exactly, but I do feel more understood. I feel more just sad than angry or upset, which I think is good because it means I feel less impulsive and more reflective. I also thought the discussion of God and religion was interesting—it was neither religious nor non-religious, but I almost wish they had picked whether to base it in spirituality or not rather than trying to relate to everyone.”
Understood (not alone, specific personal experiences, real)	4 (17.4)	“It was comforting to understand that other people could also feel what you are going through.”
Hopeful or helpful (effective or encouraging or engaging)	3 (13)	“I really liked it and thought it was good to see that someone felt hope on the other side.”
Knowledge (crisis resources or examples ways people have found help)	2 (8.7)	“I find it very knowledgeable but i would have love to here the deepest side of their stories.”
Indifferent	1 (4.3)	“The film interview is reserved and more elaborate and its okay.”

**Table 8:** Content analysis of qualitative data: respondents’ thoughts about whether they would watch the video again.

Would you watch this video again on your own time if you were going through a hard time? Why or why not?	Values, n (%)	Representative quote
Yes	8 (53.3)	“Yes as it will improve my situation.”
No	4 (26.6)	“Probably not, due to the length.”
Maybe	3 (20)	“Not sure because its long.”
Too long	3 (33.3)	“Given the sheer length of the interview, I don’t think I’d watch it if I were going through a very hard time.”
Relatable	2 (22.2)	“I would watch it again because it is like seeing your own struggles and recover through other people’s lens.”
I do not rewatch things	1 (11.1)	“Probably not. I think it’s the sort of thing that would be helpful to watch once, but I very rarely re-watch anything. I’d consider re-watching it if I needed a nice little cry or moment of understanding.”
Too close to own experience	1 (11.1)	“Maybe only some parts of it since other parts felt very close to my own experience, so the emotions from that time came up.”
Not relatable	1 (11.1)	“No, because I don’t resonate with her struggles. Her experience of recognition for academic giftedness actually made me a little embarrassed for my own attainments, which were limited in part due to my mental health spiraling pre- and post-COVID.”
Helpful or reminder of tools	1 (11.1)	“Yes. I think it would remind me of some tools available.”

When asked about overall reactions to the filmed interview, half (8/16) of the responses were coded as “positive outcome or emotion.” In total, 5 responses had specific suggestions, 4 responses were coded indicating that the filmed interviews led to them feeling more understood, 3 responses were coded as “hopeful or helpful,” 2 were coded as “knowledge,” and 1 was coded as “indifferent.” When asked if they would watch the video again if going through a hard time, over half (8/15) said yes, 4 said no, and 3 said maybe (1 respondent did not answer this question). Additional information provided was also coded with 3 responses indicating that the video was too long, 2 saying that they liked that it was relatable, 1 indicating that they do not rewatch things, 1 indicating that the video actually felt too close to their own experience, 1 indicating that it was not relatable, and 1 mentioning that it was a helpful reminder of tools.

In total, 3 responses were collected about the active control video to assess safety and acceptability; however, because of the small sample size, we cannot compare it to the “Papageno” videos. The average mood change from before to after the active control video was –0.67 (SD 0.58). All of the answers for the PFS were either agree or neutral. All of the participant answers from the AIM and IAM measuring acceptability and appropriateness were agree or neutral, except for 1 participant who responded “disagree” for “The filmed interview meets my approval.” However, this respondent also stated “nothing” when asked, “What would you change about the filmed interview?” indicating some level of acceptability and appropriateness. Participants reported feeling either somewhat more understood or no change in feeling more or less understood. Participants reported feeling somewhat or significantly more likely to reach out for help when needed. In total, 2 participants reported feeling somewhat more hopeful about the future, and 1 participant reported feeling significantly less hopeful about the future. This participant responded, “I like that fact that an emergency number is displayed to reach out anytime someone is having a suicidal thoughts” to the question “What did you like about the filmed interview?” and “good” to “What are your overall reactions to this filmed interview?” When prompted, “Please provide any additional comments or suggestions for us related to the filmed interview you watched,” one participant responded, “It is really helpful and I’m glad i watched it.” When asked, “Would you watch this video again on your own time if you were going through a hard time? Why or why not?” participants responded, “Somehow”, “Yes,” and “Yes ... I will watch.” The Cohen Identification Scale responses ranged from neutral to strongly agree, except for 1 participant who responded “disagree” to “While viewing the filmed interview, I felt as if I was there” and “While viewing the interview, I could feel the emotions the interviewee portrayed.”

## Discussion

### Principal Findings

The filmed interviews we created of young adults sharing their “Papageno” stories were generally reported to be acceptable and appropriate by our sample of 18- to 24-year-old people with a past-month history of suicidal ideation. In their qualitative feedback, respondents recommended shortening the videos and including multiple perspectives combined in 1 video. These results will guide further revisions, leading to the creation of a singular shortened video including clips from all 5 interviewees. Additionally, as a result of the feedback received, we plan to emphasize focus on the interviewee (and minimize focus on the interviewer) and preserve elements that were reported to be helpful or inspire hope.

Although we did run descriptive statistics on the data collected, the sample size is too small to capture statistical significance, and inferential conclusions cannot be drawn. The average scores on the acceptability and appropriateness scales were all higher than neutral or 3 (range 1-5), with the highest average from the PFS being “I understood the filmed interview” (mean 4.5, SD 0.52). The highest average from the AIM was “I welcome the filmed interview” (mean 4.31, SD 0.60). The highest average from the IAM was “The filmed interview seems applicable” (mean 4.13, SD 0.62). No respondents reported feeling less likely to reach out for help in the future after watching the filmed interview. Moreover, other than the 1 potentially erroneous response, no respondent reported feeling less hopeful about the future after watching the filmed interviews. These preliminary findings suggest that the Papageno story interview may show promise as an intervention for young adults with recent histories of suicidal ideation, but must be tempered by the limitations of the small sample size and uncontrolled pilot design.

The lowest average scores were for “I forgot myself and was fully absorbed” (mean 3.06, SD 1.18) and “I felt as if I was there” (mean 3.13, SD 1.06), with a score of 3 meaning “neutral.” However, other measures of identification with the interviewee were 4 or above. Helping the viewer to feel more absorbed and as if they were there might help them to connect further with the story and imagine what will happen next, which is a key component of narrative transportation that can alter attitudes [32]. Our iterative video that will be put forward for an RCT is shortened and includes clips from all 5 interviewees in order to increase the likelihood that a viewer might identify with a story and thereby increase the likelihood of narrative transportation.

It is promising that over half (n=8) of the responses indicated that participants would watch the video again on their own time if they were going through a hard time (with an additional 3 responses indicating that they might). Of the 4 responses indicating that they would not watch it again, 2 indicated that this was due to its length, 1 simply said “No,” and 1 cited that they were unable to relate to it. By combining multiple stories in 1 video, we are hopeful that more viewers would find something relatable about it. By shortening the length of the video, we are hoping that this will minimize any time commitment barrier and increase engagement.

### Comparison With Prior Work

This uncontrolled single-group pilot study supported filmed “Papageno” story interviews’ acceptability and appropriateness. The feedback received guided next steps in our pursuit toward a finalized singular intervention video combining clips from the original interviews, which could be tested for effectiveness in an RCT. A recent RCT of an “adolescent with past suicidal ideation, describing his story of getting help and recovering” showed that adolescents reported significantly lower suicidal ideation after watching the video [25]. Those who watched the video in that study also reported increased help-seeking intentions, which were maintained at 4-week follow-up. They also found a statistically significant decrease in favorable attitudes toward suicide (which is associated with increased suicide risk). After incorporating the feedback gathered from this study, the finalized intervention video we will test in an RCT (using similar methods) will differ because it will include all 5 interviewee perspectives in order to maximize the opportunity for viewer identification.

Compared with the Sources of Strength study [16], this study was a very small uncontrolled pilot from which conclusions about effectiveness or suicide outcomes cannot be made. Our study did not look at outcomes such as suicidal ideation or suicide attempts. Our study also was not an effectiveness study.

An Australian pilot study of a peer-to-peer video campaign called “Better Off With You,” which involved videos challenging the notion of perceived burdensomeness, found no changes in participants’ perceived burdensomeness, psychological distress, or help-seeking [33]. The campaign was tested in a community sample (n=157, age 20-65 years) compared to our study, which involved an eligibility criterion of past-month history of suicidal ideation. The qualitative interviews of participants indicated that people felt the campaign was safe and not distressing to people with lived experience. They concluded, “This result supports a meta-analysis that concluded that asking participants about suicide ideation or exposure to suicide-related content does not increase suicide risk” [34]. Future research is needed to understand which types of video content work best for which populations. A future direction might involve a study design where participants are allowed to choose the video they would like to watch by the preview thumbnail and short description or a study design that evaluates the real-world effectiveness of videos when presented via social media advertisements.

While each of the 5 videos we piloted featured only 1 interviewee, due to the feedback we received, the video we intend to put forward for an RCT will include all 5 perspectives, which will distinguish it from the aforementioned study. If the finalized video shows positive effects in the RCT, similar videos could be disseminated through social media or as a part of targeted interventions or therapies such as SSIs. A future direction in this field involves continued development and testing of various types of videos to further refine what elements make these videos more or less effective in their goal of suicide prevention.

### Limitations

Although consistent with a small pilot study of intervention acceptability and feasibility, a limitation of this study is that we are unable to draw any conclusions regarding the effect of the Papageno video intervention, as we did not conduct an RCT. In addition, we are unable to generalize findings to the broader population of young adults because of the small sample size and sample homogeneity. Because this was a small pilot study, we were not able to obtain a representative sample. Recruitment via Instagram and community organizations may also invite bias. Although all participants had a past-month history of suicidal ideation, we were unable to further characterize their suicide risk. Recruitment of participants with higher suicide risk might necessitate alternative recruitment methods (as investing in clicking an Instagram advertisement or engaging with a message from a community organization demonstrates some amount of baseline help-seeking). Using only Instagram and not other social media platforms may also introduce a bias related to people who use or do not use Instagram. Future studies are needed to determine the effects for individuals with both lower or higher suicide risk.

Another limitation is that participants were not queried at a follow-up time point, so any data reported are associated with the immediate postvideo time point. Because participants could watch multiple videos, it is possible that the sustained influence of the videos watched first could impact the feedback gathered from subsequent videos. At the same time, they may have found something uniquely helpful about subsequent videos.

Although missing data were rare, bias could have been introduced if people’s responses would have diverged greatly from the average response, which is especially possible if they intentionally skipped the question.

### Conclusions

The feedback received about these filmed interviews indicates that they are generally acceptable and safe. The qualitative feedback received, which pointed toward shortening the videos and including multiple perspectives, will be used to edit the 5 filmed interviews into 1 singular shortened intervention video to be put forward for an RCT. This pilot study adds momentum to the further study of “Papageno” stories shared in video format.

## Appendix

Multimedia Appendix 1 TIDieR checklist for the video interventions.

## Abbreviations

AIM: Acceptability of Intervention Measure
IAM: Intervention Appropriateness Measure
IRB: institutional review board
PFS: Program Feedback Scale
PI: principal investigator
RCT: randomized controlled trial
SSI: single session intervention
TIDieR: Template for Intervention Description and Replication
