# Supplementary material for: A “Papageno” Story Interview Suicide Prevention Intervention for Young Adults With Past-Month Suicidal Ideation: Uncontrolled Single-Group Pilot Study of Feedback and Acceptability
Source: JMIR Form Res. 2025 Oct 23;9:e71368. doi: 10.2196/71368 (PMC12592894; doi:10.2196/71368)
Supplement: Multimedia Appendix 1 [file formative_v9i1e71368_app1.pdf]

TIDieR

A “Papageno”-story Interview Suicide Prevention Intervention for Young Adults With Past Month Suicidal Ideation: Uncontrolled Single-Group Pilot Study of Feedback and Acceptability

**Why:**	Suicide protective effects can occur when alternatives to suicide are modeled and shared in media. This protective effect is often referred to as the “Papageno effect”. National leadership has acknowledged the urgent need for creative collaborations including leveraging the "Papageno effect" in suicide prevention strategies.
**What (material):**	The piloted interventions include five filmed interviews (ranging in length from 20-35min each) of young adults, ages 18 to 24 years, with personal histories of finding non-suicide alternatives (“Papageno” stories). Each interviewee was asked questions about a time in their life when they came close to suicide and then found non-suicide alternatives. Additionally, the interviews focused on recovery, help-seeking, reasons for living and hope for the future.
**What (procedures):**	Eligible participants competed pre- and post- intervention questionnaires from which quantitative and qualitative data was collected and analyzed. They were not able to advance past the video until at least half the time of the video had elapsed (in case they decided to watch it on 2x).
**Who provided:**	The intervention is web-based and self-guided.
**How (mode of delivery; individual or group):**	The intervention is web-based and self-guided. Participants watch the video individually on their own time.
**Where:**	This is a web-based intervention that was hosted on Qualtrics (videos were embedded into the surveys).
**When and how much:**	This was a one time intervention.
**Tailoring:**	Each participant could ask to give feedback on up to 5 videos. The intervention was not personalized or titrated. Participants had no choice in which videos they were given.
**Modification:**	The intervention videos were not modified during the course of the study.
**How well (planned):**	It is not possible to know if the participants watched the video in its entirety or how attentive they were to the video, however we did design the survey so that they could not advance to the next page until at least half the time of the video had passed (in case they decided to watch it on 2x). For some participants, the level of detail of the qualitative feedback that we collected is indicative of them actually watching the video(s).
**How well (actual):**	There were only 3 instances of missing responses for our quantitative questions.
